# ToxCast^™^ Wants You: Recommendations for Engaging the Broader Scientific Community

**DOI:** 10.1289/ehp.123-A20

**Published:** 2015-01-01

**Authors:** Carrie Arnold

**Affiliations:** Carrie Arnold is a freelance science writer living in Virginia. Her work has appeared in *Scientific American*, *Discover*, *New Scientist*, *Smithsonian*, and more.

Since the U.S. Environmental Protection Agency (EPA) launched ToxCast™ in 2007, this chemical screening program has generated massive amounts of data. The main objective of ToxCast has been to help the agency prioritize chemicals for further review to meet different regulatory needs. But ToxCast may have much more to offer the broader research community—and the broader research community has much to offer ToxCast. A new commentary in this issue of *EHP* discusses two strategies for increasing engagement between ToxCast and researchers in disciplines beyond toxicology.^1^

Under the Toxic Substances Control Act, the EPA maintains an inventory of chemicals produced and processed in the United States. There are currently more than 84,000 chemicals on the inventory, and 500–1,000 additional chemicals are added each year.^2^

**Figure:**
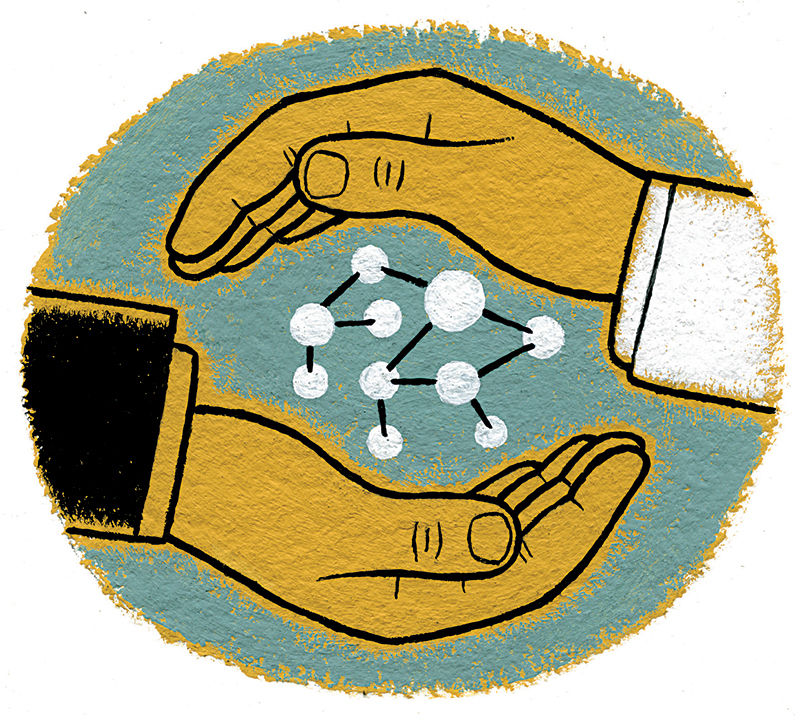
ToxCast and the broader research community have much to offer one another on the quest to better understand the chemicals we use. © 2014 James Steinberg c/o theispot.com

But for most of these chemicals there are few or no toxicity data, and traditional toxicity testing is slow and expensive.^3^ Relying on traditional approaches alone, it would take decades to evaluate the tens of thousands of chemicals that lack adequate data to support regulatory action. “Concern is great that among the untested chemicals in wide use there may lurk currently undiscovered human toxicants,” says Philip Landrigan, a pediatric researcher at the Mount Sinai School of Medicine, who was not involved in the commentary.

The Tox21 consortium—which also includes the National Toxicology Program (NTP), the National Center for Advancing Translational Sciences, and the Food and Drug Administration—is a federal initiative aimed at focusing and speeding up this discovery process.^4^ ToxCast, short for Toxicity Forecaster, is one of the EPA’s main contributions to the Tox21 collaboration. ToxCast uses high-throughput *in vitro* screening to flag compounds that show signs of potential toxicity. These compounds are then prioritized for more in-depth study.^5^

In the new commentary, Jennifer McPartland, a health scientist at the Environmental Defense Fund, and colleagues suggest new approaches to broaden scientific engagement with ToxCast in particular and Tox21 overall. “We need more researchers to engage with the emerging data so that we are ultimately making better public health decisions,” McPartland says.

The authors point out that making better decisions depends in large part on the scientific integrity of the assays used in ToxCast (and other high-throughput *in vitro* initiatives) and the scientifically sound interpretation of the data those assays produce.^1^ This is where the larger community comes in. Kristina Thayer, director of the NTP Office of Health Assessment and Translation, explains, “The broader research community is well poised to do the orthogonal testing required to assess the utility of ToxCast predictions, which is needed to help with regulatory acceptance.” By “orthogonal testing,” she means the use of a different assay—usually one that is closer to the target physiological condition or using a different technology—to assess ToxCast results. (Thayer was not involved with the commentary.)

McPartland and colleagues first recommend using collaborative workshops to introduce ToxCast to a wider scientific audience.^1^ As an example of how this might work in practice, they point to a 2011 workshop conducted by the NTP in which experts from a spectrum of fields assessed the scientific literature and ToxCast data related to the role of chemical exposures in obesity and diabetes.^6^ The workshop gave NTP staff a chance to explain ToxCast to these researchers, who in turn provided expert analysis and feedback on the data produced by ToxCast. After the workshop, the NTP teamed up with some of the participants to discuss priority chemicals identified by ToxCast, which those participants might study in their own laboratories.^1^

Their second recommendation is to seek out mutually beneficial research partnerships like one established between Harvard and the EPA.^1^ Russ Hauser, an epidemiologist at Harvard School of Public Health, learned about ToxCast data when he served on an EPA Science Advisory Board. Hauser had been studying children with very early onset inflammatory bowel disease (VEO-IBD), which includes diseases such as Crohn’s disease and ulcerative colitis that are diagnosed in children under age 10.^7^ Together with EPA scientists, pediatric gastroenterologists, and pediatric immunologists, Hauser is co-leading a project using ToxCast data to identify environmental factors that may contribute to VEO-IBD.

“There’s no way we could measure the effects of dozens of chemicals in a human study,” Hauser says. “Our work wouldn’t be possible without ToxCast data.”

Tina Bahadori, director of the EPA’s Chemical Safety for Sustainability research program, says the agency has already been thinking along the same lines as McPartland and colleagues and is working toward implementing these recommendations. “This is exactly the feedback that we’re looking for,” says Bahadori, who was not involved with the commentary. “Not only is it useful, it also gives us the justification that’s needed to broaden our landscape and look at environmental and public health applications of these data from a broader vantage point than what we’re accustomed to.”

## References

[1] McPartland J Building a robust 21st century chemical testing program at the U.S. Environmental Protection Agency: recommendations for strengthening scientific engagement. Environ Health Perspect 123 1 1 5 2015; 10.1289/ehp.1408601 25343778 PMC4286280

[2] EPA. TSCA Chemical Substance Inventory [website]. Washington, DC:U.S. Environmental Protection Agency (updated 13 March 2014). Available: http://www.epa.gov/oppt/existingchemicals/pubs/tscainventory/basic.html [accessed 4 December 2014]

[3] Judson R The toxicity landscape for environmental chemicals. Environ Health Perspect 117 5 685 695 2009; 10.1289/ehp.0800168 19479008 PMC2685828

[4] NTP. Tox21 [website]. Research Triangle Park, NC:National Toxicology Program, National Institute of Environmental Health Sciences, National Institutes of Health (updated 2 December 2014). Available: http://ntp.niehs.nih.gov/results/hts/index.html [accessed 4 December 2014]

[5] EPA. Toxicity Forecaster (ToxCast™) [fact sheet]. Washington, DC:U.S. Environmental Protection Agency (undated). Available: http://www.epa.gov/ncct/download_files/factsheets/Tox_Cast_Fact_Sheet.pdf [accessed 4 December 2014]

[6] Thayer KA Role of environmental chemicals in diabetes and obesity: a National Toxicology Program workshop review. Environ Health Perspect 120 6 779 789 2012; 10.1289/ehp.1104597 22296744 PMC3385443

[7] Benchimol EI Incidence, outcomes, and health services burden of very early onset inflammatory bowel disease. Gastroenterology 147 4 803 813 2014; 10.1053/j.gastro.2014.06.023 24951840

